# Student outcomes in a student-led clinic: beyond clinical learning

**DOI:** 10.3389/frhs.2026.1884776

**Published:** 2026-10-09

**Authors:** P. Broman, S. Vaughan, P. Andersen, M. J. Cannings, M. B. P. Haggie, K. O’Connor, K. Kovanur Sampath, S. Tan, O. Webster, J.-R. Yap, S. Brownie

**Affiliations:** 1 Australian Centre for Student Equity and Success, Curtin University, Perth, WA, Australia; 2 Centre for Health and Social Practice, Wintec, Hamilton, New Zealand; 3 Social Impact Team, Huber Social, Sydney, NSW, Australia; 4 School of Health, University of the Sunshine Coast, Maroochydore, QLD, Australia; 5 Te Wānanga Waiora | Division of Health, University of Waikato, Hamilton, New Zealand; 6 School of Health and Biomedical Sciences, RMIT, Melbourne, VIC, Australia; 7 Centre for Osteopathic Research and Leadership (CORAL), Health Sciences University, London, United Kingdom; 8 Ravi Nair College of Physiotherapy, DMIHER (DU), Maharashtra, India; 9 Social Impact Team, Huber Social, Perth, WA, Australia; 10 English Language Partners New Zealand, Welington, New Zealand; 11 School of Rural Medicine, Charles Sturt University, Orange, NSW, Australia

**Keywords:** health professions education, learning outcome, student-assisted clinic, student-led clinic, wellbeing

## Abstract

**Introduction:**

Student-assisted, -run or -led health clinics (SLCs) are increasingly common worldwide, but student outcomes remain under-evaluated, with existing work focused narrowly on clinical and/or academic outcomes.

**Methods:**

This study presents the findings of an evaluation of the impact of a student-assisted clinic in New Zealand, on participating students subjective wellbeing, assessed using the Satisfaction with Life Scale (SWLS), as well as on a range of programme-related intermediate outcomes theorised to support wellbeing, including leadership, communication, and clinical capability. Students undertaking placements at the clinic were surveyed at three time points: at the start, the end, and at six months post-placement.

**Results:**

Participating students maintained levels of self-reported wellbeing over placement periods while significantly improving leadership, purpose, cultural connection, and clinical capability. Income and sleep were shown to be key factors related to SWLS scores.

**Discussion:**

Student-related outcomes from SLCs may extend beyond clinical care and academic performance, highlighting the value of a more holistic approach to SLC evaluation.

## 1 Introduction

Understandings of “success” in higher education increasingly extend beyond academic attainment to encompass broader developmental outcomes, including student wellbeing. Wellbeing—conceptualised as a multidimensional construct including emotional, psychological, and social dimensions—has been shown to underpin engagement, resilience, intrinsic motivation, and longer-term educational and professional outcomes (1, 2). Alongside wellbeing, constructs such as cultural competence, leadership, and purpose are central to emerging frameworks of transformative education supporting identity formation, civic responsibility, and sustained motivation (3–5).

Despite their recognised importance, wellbeing outcomes remain infrequently and inconsistently assessed within higher education (6, 7). Prevailing evaluation and assessment regimes continue to privilege proximate, quantifiable academic indicators, such as grades, completion rates, and technical competencies over complex, longitudinal, and context-dependent constructs such as wellbeing or purpose (8, 9). This tendency is particularly evident in health professional education, where student assessment has historically focused on clinical or technical competence, often at the expense of broader social competencies (10). Social competencies encompass a range of interpersonal and relational capabilities, including communication, teamwork, leadership, empathy, cultural responsiveness, and the ability to work effectively with others in complex healthcare environments, all now well-recognised as critical elements of modern healthcare practice (11–14). Such competencies have been linked to wellbeing, directly inasmuch as they support improved social connectedness and professional fulfilment (5, 15), and indirectly for healthcare workers through enhanced confidence, belonging, and effectiveness within clinical teams (11, 16, 17).

These evaluative limitations extend to evaluations of student-led, student-run or student-assisted health services or clinics, which continue to proliferate internationally (18, 19). Such services provide a mechanism for health professional students to gain authentic clinical experience during their educational programme, and/or provide care to underserved communities (20). While the terms are often used interchangeably, because such models share the common characteristic of students participating in real-world service delivery, important distinctions exist. In student-assisted clinics, students typically contribute healthcare within a service led and governed by qualified practitioners. Student-led clinics suggest students take a more active role in directing patient care and coordinating service delivery, while student-run clinics may additionally involve students in operational management, governance, or organisational functions (18).^1^ While recognising the important differences in student responsibility, autonomy, and governance, where discussing the broader evidence base, we use “student-led clinic” as a collective term to encompass this family of student-engaged clinical service models.

Evaluation of such services, to date, has concentrated largely on patient health outcomes (21, 22), costs (23, 24), and students’ self-reported clinical learning or satisfaction (25, 26). Scholars have repeatedly noted that this literature is fragmented and methodologically narrow, with limited use of integrated frameworks capable of capturing the multi-faceted goals and impact of student-assisted clinics as both educational environments and sites of community care (19, 27). Although a small body of work has examined adjacent constructs such as leadership development, professional identity, empathy, or commitment to underserved populations (28–30), these studies rarely operationalise wellbeing explicitly or employ validated wellbeing measures. As a result, impacts of student-led clinic involvement on students’ broader psychosocial development, and particularly their wellbeing, remain poorly theorised and understood.

This study addresses this conceptual and empirical gap by positioning student wellbeing as a central outcome in the evaluation of a student-assisted health clinic in New Zealand. The clinic's Theory of Change identified subjective wellbeing as an ultimate outcome sought for participating students. It was hypothesised that wellbeing would be influenced through a series of intermediate outcomes, including leadership development, community connection, cultural understanding, clinical capability, and sense of purpose. These outcomes were conceptualised as key mechanisms through which participation in the clinic could contribute to students’ broader psychosocial development and capacity to thrive both during their studies and in their future professional practice. This study examines changes in subjective wellbeing alongside these intermediate outcomes to better understand the extent to which participation in a student-assisted, interprofessional health clinic is associated with student development and wellbeing. By integrating wellbeing theory and measurement into the evaluation of student-assisted health services, the study contributes to emerging discussions about holistic impact assessment in health professional education and reframes such clinics as developmental learning environments whose value extends beyond as vehicles for clinical skill acquisition and academic achievement alone.

## 2 Methods

### 2.1 Setting

*Te Kotahi Oranga* was established in 2022 as a student-assisted health clinic at Waikato Institute of Technology, a major vocational education provider in Hamilton, New Zealand. The development of the clinic followed extensive exploratory work by staff assessing local healthcare need and feasibility (31). The clinic involved students from nursing, occupational therapy, social work, clinical exercise physiology, and other disciplines, with placement duration varying by programme requirements and stage of study. Students undertook health screening, assessment, health education, falls prevention, pre-diabetes management, and other community-based health interventions, and focussed on interventions targeting type 2 diabetes, COPD, adult asthma, and fall-risk prevention—areas prioritized based on analysis of health sector need and guidance from partner community organisations (32). Care was delivered through a combination of clinic-based appointments in a dedicated space on campus and, where appropriate, community or home visits. Students actively participated in client assessment, care planning, health promotion activities, interprofessional case discussions, and follow-up care; however, clinical governance and responsibility remained with qualified health professionals.

Placements at *Te Kotahi Oranga* differed from traditional student placements in that it gave a unique opportunity for students to gain early, team-based clinical and leadership experience using culturally informed models of care. The project was conceived and operated as a close collaboration with local health and social sector organisations, including Māori and Pacific providers, and delivered culturally responsive care embedded within Te Tiriti o Waitangi principles.^2^ It received significant financial backing from local philanthropic organisations, which funded a purpose-built facility, while the associated education provider gave in-kind and staffing support.

From the outset, while seeking to provide a health service, the clinic also aimed to improve the overall wellbeing of participating students. Its Theory of Change posited that student wellbeing would be improved by fostering leadership, connection, cultural understanding, and purpose, which were identified as key pathways to students fulfilling their potential.

### 2.2 Study design

This study employed a prospective, longitudinal evaluation design to examine changes in student wellbeing and programme-related outcomes associated with participation in a student-assisted health clinic. Data were collected at three time points: (1) baseline, within the first two weeks of placement (*n* = 64); (2) post-placement, within two weeks of placement completion (*n* = 44); and (3) six months following placement completion (*n* = 16). This design was adopted to allow assessment of both short-term outcomes and longer-term impact. A total of 124 surveys were completed, with 64 at baseline, 44 within two weeks post-placement and 16 six months post-placement.

The survey contained 57-items and was undertaken online using the *Alchemer* platform. Responses were matched over time periods via a question in the survey which asked for unique Student Identification numbers. Students indicated the time period via a question asking “When are you completing this survey?” and gave the options of: “At the start of your placement at Te Kotahi Oranga”, “At completion of your placement at Te Kotahi Oranga”, or “Approximately six months after your placement at Te Kotahi Oranga”. Links to the survey were provided in-person by programme staff during orientation and during a debrief exercise (for baseline and post-placement data collection). Six month follow-up data collection was via an email sent directly to students institutional email address. As some students did not complete the placement, response rates cannot be determined.

### 2.3 Participants

All participating students were invited to take part in data collection. A total of 73 students completed at least one survey between October 2022 and November 2024. Table 1 shows key participant information at baseline (*n* = 64).

**Table 1:** Participating students^a^.

Sex	*n*	%
Female	48	75%
Male	15	23.4%
Did not answer	1	1.6%
Total	64	100%

Age	*n*	%
18–24 years	27	42.2%
25–34 years	24	37.5%
35–44 years	8	12.5%
45–54 years	3	4.7%
Did not answer	2	3%
Total	64	100%

Cultural Heritage^b^	*n*	%
New Zealand European	22	34%
Māori	13	20%
Samoan	1	2%
Cook Island Māori	2	3%
Tongan	1	2%
Niuean	1	2%
Indian	18	28%
Another ethnicity	16	25%
Total	64	100%

a Measured at baseline (*n* = 64).

b Participants could select up to two cultural heritage groups so that responses do not sum to 100%.

Participants were primarily studying Nursing or Occupational Therapy, with 95% coming from these disciplines, though students from clinical exercise physiology, social work, massage and hospitality programmes were also involved. Respondents were mostly in their first (*n* = 37, 60%) or second (*n* = 22, 35%) year of study. 31 (50%) of participants had 0 dependents, 13 (21%) 1 dependent, 12 (19.4%) 2 dependents, and 6 (10%) 3 or more dependents.

### 2.4 Measures

A participatory process was used during the development of the evaluation framework and survey instrument. This process involved academic staff, clinic representatives, and members of the impact evaluation consultancy *Huber Social* in identifying the outcomes considered most relevant to the clinic's objectives. The collaborative design process informed the selection of outcome domains and the operationalisation of survey measures.

The resulting evaluation framework focused on subjective wellbeing as the primary outcome, measured using the validated *Satisfaction with Life Scale* (SWLS), a widely used five-item measure of global life satisfaction (33). The SWLS does not capture all dimensions of wellbeing, but provides a validated measure of individuals’ overall evaluation of their lives, consistent with established wellbeing research. The survey also included several other measures relevant to holistic wellbeing, such as physical health, mental health, personal relationships, access to resources, and sleep quality. These measures were included because subjective wellbeing is influenced by a broad range of factors both within and beyond educational settings. While it was not assumed that clinic participation would directly affect these domains, they were measured to provide a more comprehensive understanding of students’ wellbeing and to identify factors associated with variations in life satisfaction.

No existing instrument was identified that adequately captured the more proximal educational, cultural, interprofessional, and community-engagement outcomes sought and articulated within the clinic Theory of Change as contributing to wellbeing. Programme-specific survey items were developed to operationalise constructs related to clinical leadership, cultural understanding, interprofessional collaboration, clinical capability, connection, and purpose in ways that were contextually relevant to the student-assisted clinic environment and aligned with the programme's objectives. These items were not designed as psychometric subscales intended to measure latent constructs through multiple interchangeable indicators. Rather, they were developed as individual outcome indicators aligned with the clinic's Theory of Change and intended to capture specific programme-related outcomes considered important by stakeholders. Consequently, internal consistency statistics were not calculated, and the items were analysed and interpreted individually rather than combined into composite scales.

Draft items were reviewed by stakeholders for clarity, face validity, cultural appropriateness, and relevance to the educational setting before being incorporated into the final survey instrument. These outcomes were assessed through a series of Likert-type items (1 = Completely disagree to 7 = Completely agree).

### 2.5 Analysis

Quantitative data were analysed using both paired-sample and group-level statistical approaches, depending on the availability of matched responses. The analysis included descriptive statistics, tests of statistical significance, and correlation analyses. Pearson and Spearman correlation coefficients were used to identify correlations with SWLS measures, with significance thresholds set at *p* < .10 for exploratory trends worthy of further study and *p* < .05 for core inferential results. These analyses enabled the identification of both programme-influenced outcomes (e.g., leadership, cultural understanding) and broader determinants of wellbeing (e.g., income sufficiency, sleep).

Statistical assumptions were assessed prior to inferential testing. Normality was assessed using the Shapiro–Wilk test, with a non-significant result (*p* > .05) indicating that the normality assumption was not violated. For paired analyses, where the relevant normality assumption was not met, the Wilcoxon signed-rank test was used instead of the paired-samples *t*-test. For independent-samples analyses, where the relevant normality assumption was not met, the Mann–Whitney *U* test was used.

The study sample was determined by the available student cohorts, rather than through a formal *a priori* sample-size calculation. The intention was to obtain sufficient paired observations to approach 80 percent statistical power. Baseline to post-placement comparisons were conducted using 32 paired cases, providing moderate estimated *post-hoc* power of 0.782. However, participant attrition, particularly at the six-month follow-up, reduced the number of complete matched longitudinal observations available for analysis. For the six-month comparisons, paired analysis provided *post-hoc* power estimates of 0.353. Group-level independent analysis was therefore undertaken, with an estimated *post-hoc* power of 0.424. Power estimates describe the statistical sensitivity of the analyses given the final available samples, rather than as the basis for selecting the statistical tests.

Missing data were handled using complete-case analysis (case deletion), and no imputation of missing values was performed. No formal adjustment for multiple comparisons was applied, given the multiple outcomes examined and the relatively modest sample size. The study examined a range of outcomes identified in the clinic's Theory of Change, with analyses considered exploratory in nature. Consequently, individual *p*-values should be interpreted cautiously, as conducting multiple statistical tests increases the risk of Type I error (false-positive findings).

### 2.6 Ethics

The evaluation was approved by Waikato Institute of Technology Ethics Committee (approval No. WTFE10111022). All student participants were briefed on the objectives of the research and their rights and provided written consent to participate.

## 3 Results

The primary targeted impact of the clinic for students was subjective wellbeing. Global life satisfaction was assessed using the Satisfaction with Life Scale (SWLS), as an indicator of subjective wellbeing. Most students reported moderate to high subjective wellbeing at all time points. Mean SWLS scores increased slightly between baseline and post-placement measurement (+4%) before returning close to baseline levels at 6-month follow-up; however, these differences were not statistically significant. Given the very small sample size, findings from the six-month follow-up should be interpreted with caution. While overall life satisfaction scores were similar at the group level, the six-month respondent group reported higher scores than the baseline group on several programme-specific outcomes, including leadership, community connection, communication, and clinical capability.

Correlation analysis was conducted to identify the wellbeing-related factors associated with participating students’ SWLS scores (Table 2). These variables were measured to provide contextual information about students’ broader circumstances and to identify factors associated with global life satisfaction. Correlation analyses between these indicators and SWLS scores were undertaken to explore which factors were most strongly related to students’ life satisfaction and to distinguish programme-related outcomes from broader influences operating outside the clinic's sphere of control. The strongest association with life satisfaction was observed for having sufficient income to meet everyday needs (*r* = 0.612, *p* < 0.001). Moderate positive associations were also observed between SWLS scores and sleep sufficiency, access to appropriate career development opportunities, strong personal support networks, and motivation to work with underserved communities.

**Table 2:** Correlation between SWLS and selected indicators.

Indicator	Pearson's *r*	*p*
I have enough income to cover my everyday needs	0.612	<.001
On most days I get enough sleep and feel well rested	0.55	<.001
The career development opportunities I have access to are appropriate for my career goals and interests	0.543	<.001
I have a strong support network of family, whānau^a^ and/or friends	0.522	<.001
I feel motivated to pursue a career with underserved communities	0.493	<.001

a Whānau is an Indigenous Māori term denoting extended family in a broad sense, including physical, emotional and spiritual dimensions and community.

Within several wellbeing-related capability domains, identified in the Theory of Change as secondary outcomes, paired analysis of baseline and postplacement responses (*n* = 32) demonstrated statistically significant increases (Table 3). Scores were compared using paired-samples *t*-tests where the assumptions for parametric testing were met. Paired analyses were used because sufficient matched baseline and post-placement observations were available. Where the assumptions for the paired-samples *t*-test were not met, the Wilcoxon signed-rank test was used as the non-parametric alternative.

**Table 3:** Short-term outcomes (baseline vs. post-placement)^a^.

Factor	Baseline mean	SD	Follow-up mean	SD	Mean difference (baseline—follow-up)	95% CI for location parameter	Effect size	Statistical test	*p* value
I have had the opportunity to lead client care	4	1.7	6.19	0.9	−2.19	[−2.83, −1.44]	*d* *=* *−1.14*	Paired t, t (29) = −6.25	<.001
Through my clinical work, I feel a sense of connection with the community	5.09	1.2	6.28	1	−1.19	[−2.00, −1.00]	*rrb = -0* *.* *83*	Wilcoxon, *W* = 30, *z* = −3.70	<.001
When appropriate, I am comfortable leading a team of my peers in client care management	4.75	1.4	5.88	1	−1.13	[−2.00, −1.00]	*rrb = -0* *.* *79*	Wilcoxon, W = 39, *z* = −3.60	<.001
Within my clinical team, I am able to advocate for myself and my client	5.31	1.1	6.44	0.7	−1.13	[−2.00, −1.00]	*rrb = -0* *.* *85*	Wilcoxon, *W* = 28.5, *z* = −3.86	<.001
I am able to make decisions about client care confidently and effectively	5	1.4	5.78	1	−0.78	[−2.00, −0.50]	*rrb = -0* *.* *65*	Wilcoxon, *W* = 44.5, *z* = −2.66	0.007
I have had access to learning opportunities which allowed me to develop my clinical knowledge in community care	5.19	1.6	5.97	1	−0.78	[−1.60, −0.01]	*d* *=* *−0.37*	Paired t, t (30) = −2.07	0.047
I am confident in my ability to communicate effectively with clients and their whānau	5.34	1	6.1	0.9	−0.76	[−1.50, −0.50]	*rrb =* −*0.62*	Wilcoxon, *W* = 48, *z* = −2.55	0.01

a Data analysis used 32 paired responses (baseline vs. post-placement). All measurements are on a scale of 1–7, where 1 = Completely Disagree and 7 = Completely Agree.

Students reported increased experience in leading client care, increased comfort in leading peers within interprofessional teams, and increased self-advocacy within clinical settings. Significant increases were also observed for confidence in clinical decision-making, opportunities to develop clinical knowledge, and communication skills with clients and their families. Reported connection to the community served by the clinics also increased over the placement period.

Attrition at the six-month follow-up substantially reduced the number of participants with matched baseline and six-month observations, limiting the suitability of paired analysis. Therefore, for the seven long-term outcomes, baseline and six-month post-placement (*n* = 16) scores were compared at group-level, using independent-samples *t*-tests where assumptions were met (Table 4). As assumptions for parametric independent-samples testing were not met, the Mann–Whitney *U* test was used as a non-parametric alternative.

**Table 4:** Long-term outcomes (baseline vs. 6 months post-placement)^a^.

Factor	Baseline mean	SD	Follow-up mean	SD	Mean difference (baseline—follow-up)	95% CI for location parameter	Rank-biserial correlation (rrb)	Statistical test (Mann–Whitney *U*)	*p* value
I have had the opportunity to lead client care	4.19	1.6	5.2	1.7	−1.01	[−2.000, −1.243 × 10⁻^4^]	*−0*.*41*	263.5	0.015
Through my clinical work, I feel a sense of connection with the community	5.27	1.3	6.13	1	−0.86	[−2.000, −7.469 × 10⁻^5^]	*−0*.*38*	311.5	0.016
I am confident in my ability to communicate effectively with clients and their whānau	5.5	1.1	6.06	0.9	−0.56	[−1.000, 5.606 × 10⁻^5^]	*−0*.*28*	366.5	0.07
My clinical work gives me a sense of purpose	5.71	1.2	6.25	0.9	−0.54	[−1.000, 3.338 × 10⁻^5^]	*−0*.*25*	376	0.105
I consider the experiences and perspectives of my clients when interacting with them	5.97	0.9	6.44	1	−0.47	[−1.000, −4.425 × 10⁻^5^]	*−0*.*33*	335.5	0.03
I have had access to learning opportunities which allowed me to develop my clinical knowledge in community care	5.25	1.4	5.69	1.2	−0.44	[−1.000, 3.720 × 10⁻^5^]	*−0*.*18*	418.5	0.245
I feel motivated to pursue a career in my healthcare field	6.14	1.1	6.53	0.9	−0.39	[−1.000, 2.814 × 10⁻^5^]	*−0*.*25*	354.5	0.102

a Group-level analysis of 64 baseline responses and 16 longitudinal responses. All measurements are on a scale of 1–7, where 1 = Completely Disagree and 7 = Completely Agree.

While findings should be interpreted cautiously given the small longitudinal sample size, risk of response bias and group-level (and therefore only exploratory) analyses, amongst the 6-month follow-up group statistically significant increases (*p* < .05) were reported in leadership opportunities, community connection, and culturally attuned interactions with clients and their whānau. Additional changes, including improvements in communication skills and sense of purpose, approached but did not meet conventional levels of statistical significance, further highlighting the exploratory nature of these trends.

## 4 Discussion

These findings have implications for the design, delivery, and evaluation of health professional education, particularly where student wellbeing is an explicit or implicit objective. While overall Satisfaction with Life Scale scores remained relatively stable between the baseline to post-placement periods, even stability in SWLS scores across placement is notable, given the substantial evidence that clinical training placements are associated with declining wellbeing, elevated stress, and increased risk of burnout (34, 35). Moreover, while participation in the clinic was not associated with statistically significant changes in global life satisfaction, it was associated with improvements in several intermediate outcomes hypothesised to contribute to wellbeing, including leadership, community connection, culturally attuned practice, and sense of purpose. Several outcomes, including leadership, culturally attuned practice, and sense of purpose, were reported at higher levels by the 6-month respondent group than by the baseline respondent group. These findings are consistent with the possibility that participation in the clinic may support aspects of student development hypothesised to support wellbeing.

Several of the programme-specific outcomes that improved over the placement period, including leadership, communication, community connection, self-advocacy, and culturally attuned interactions with clients and their whānau, align with capabilities commonly described in the literature as social competencies: interpersonal and relational capabilities that enable healthcare professionals to work effectively with patients, communities, and other professionals, including communication, teamwork, leadership, cultural responsiveness, and collaborative practice (10–13). These capabilities are increasingly recognised as important for effective contemporary healthcare practice because they support patient-centred care, interprofessional collaboration, teamwork, and responsiveness towards increasingly diverse communities (12, 14, 16). The improvements observed in these programme-specific indicators suggest that student-assisted clinics may have a role to play in contributing to the development of capabilities extending beyond technical or clinical competence, consistent with evidence that authentic, community-based and interprofessional learning environments can foster broader professional and social capability development (26, 28, 30).

Participants who completed the survey 6 months post-placement reported high levels of purpose and motivation to pursue careers in healthcare. Purpose and meaning are widely recognised as central components of (fundamentally eudaimonic) wellbeing (5, 36), and have been definitively linked to improved resilience, lower psychological distress, and reduced mortality risk (15, 37, 38). The higher reported sense of purpose amongst this group compared to the baseline group may suggest that community-embedded clinical placement experiences can contribute to how students perceive the meaning and value of their work. However, because purpose was assessed using a programme-specific measure rather than a validated measure, these findings should be interpreted as indicative rather than definitive evidence of change in deeper dimensions of wellbeing that extend beyond transient affect or satisfaction.

Self-reported income security was the factor most strongly associated with students’ reported life satisfaction. Income security was well outside the clinic initiative's remit but reinforces a growing body of literature that highlights the limits of educational interventions in addressing wellbeing without parallel attention to structural and material conditions (39, 40). Fundamentally, students must have access to sufficient resources and basic security to maximise the outcomes of educational opportunities, including placements (41–43). There was also an association between income sufficiency and motivation to work with underserved communities (Pearson's *r* = 0.307, *p* = 0.014). While the direction of this relationship cannot be determined from the present data, this finding may warrant further investigation regarding whether financial factors are related to students’ career intentions and workforce aspirations. These findings further support calls (41, 44) for students to be financially supported while engaged on required learning placements.

Self-reported sleep quality was a further factor associated with SWLS scores, reflective of the now well-established relationship between sleep and life satisfaction (45–47). However, studies exploring the role of self-reported sleep adequacy on student's wellbeing are limited [for exceptions, see (48–50)]. A wider body of work demonstrates higher education students at particularly high risk for poor sleep health (51). This relationship invites further consideration of the importance of sleep health for students, including those involved in educational interventions such as this one, but also demonstrates how, as Nowlin et al. (52) have argued, campuses represent an underused locale to improve population health through sleep health promotion activity. Future research could explore whether student-assisted clinics represent a suitable setting for sleep health education or promotion initiatives.

To our knowledge, relatively few studies have explicitly examined student wellbeing as a central outcome in evaluations of student-led clinics. Existing evaluation of student outcomes for such clinics have concentrated on educational outcomes, particularly students’ acquisition of clinical skills, confidence, and readiness for practice (25, 53). A far smaller body of work extends beyond clinical competencies to examine leadership development (28), professional roles or identity formation (29, 54), and students’ commitment to or intention to work with underserved populations (30, 55). While these studies implicitly engage with constructs related to purpose or values, they have not systematically operationalised wellbeing, nor have they employed validated wellbeing indicators to examine how participation in student-led clinics intersects with students’ wider, non-professional lives.

This work addresses a clear conceptual and empirical gap by integrating wellbeing theory and measurement directly into the analysis of a student-assisted clinic model. Research on student wellbeing in higher education and training has consistently shown that wellbeing is shaped by the interaction of structural conditions, psychological capabilities, and meaningful engagement, and that learning environments can either buffer or exacerbate wellbeing risks (5, 35, 56). Yet this literature has developed largely in parallel to research on student-led clinics, with little cross-fertilisation between the two. By explicitly examining subjective wellbeing alongside related dimensions such as purpose, this study reframes student-led clinics not solely as sites for skill acquisition, but as a potential contributor to students’ broader psychosocial outcomes.

### 4.1 Limitations

While this study is the first to consider the impact of student led clinic involvement on student wellbeing, several limitations should be considered when interpreting findings. First, the study relied primarily on self-reported survey data. There appears to be no validated instruments specifically geared toward student-run clinics (57). While validated instruments were used where possible (e.g., the Satisfaction with Life Scale), self-report data are subject to response biases, and these factors may influence self-reported changes.

Second, the study employed a single-site design within one student-assisted clinic in New Zealand. The findings therefore reflect the context, population, and operational model of this clinic and may not be transferable to other student-assisted, student-led, or student-run clinics, or to other health professional education settings.

Although the evaluation employed a longitudinal component, the longitudinal sample size was very small compared to the baseline samples (*n* = 16). The reduction in responses over successive waves is typical of longitudinal survey research (58) but should be considered when interpreting findings, particularly those relating to longer-term outcomes. This has reduced statistical power at the 6-month follow-up, increased the risk of non-response bias, and means that longer-term findings can only be interpreted as indicative. Future research would benefit from larger longitudinal samples.

In addition, although the clinic operated as an interprofessional environment and included students from multiple disciplines, the participant sample was overwhelmingly drawn (95%) from nursing and occupational therapy. Findings may not be fully transferable to students from other health professions and future research should seek to include larger and more diverse samples across a broader range of health professions to assess the generalisability of these findings.

Subjective wellbeing was operationalised primarily through the Satisfaction with Life Scale, which measures global life satisfaction. While widely validated and commonly used within wellbeing research, the SWLS does not capture all dimensions of subjective wellbeing and these results should not be interpreted as such.

Finally, the observational design limits causal inference. Associations identified between indicators should not be interpreted as evidence of causal relationships. Similarly, while improvements were observed in several intermediate outcomes, the study cannot establish that participation in the clinic directly caused these changes.

## 5 Conclusion

Student-led health services can deliver significant health (21) and educational benefits (25) but straddling both healthcare and educational domains involves complicated funding structures and often dooms long-term viability. This underscores the need for evaluations of such services to capture the broadest possible outcomes, across health, educational, and community dimensions, so that educators, funders, and policymakers can make informed judgments about their true effectiveness and long-term value. This paper is to our knowledge the first to consider the impact on student subjective wellbeing as a core element in the impact and effectiveness of student-led clinics.

This article contributes to the limited literature examining student wellbeing as a central construct in the evaluation of student-led clinics. The findings, including that student placements in one such clinic was associated with improved levels of self-reported purpose, connection, and leadership over the placement period, suggest that aspects of student wellbeing can usefully be incorporated into impact assessment frameworks to enable a more complete understanding of how student-led clinics function as developmental environments. Recognising wellbeing as both an outcome and an enabling condition aligns evaluation with contemporary views of educational quality, social impact, and sustainability, and further strengthens the case for student-led clinics as learning environments that may support not only skill acquisition but the formation of capable, resilient, and thriving future health professionals.

## Data Availability

The raw data supporting the conclusions of this article will be made available by the authors, without undue reservation.

## Footnotes

^1^The clinic examined in this study is best characterised as a student-assisted clinic, as students delivered care within a supervised model in which leadership and clinical accountability remained with qualified staff.

^2^Te Tiriti o Waitangi (the Treaty of Waitangi) is New Zealand’s founding document, signed in 1840 by representatives of the British Crown and Indigenous Māori chiefs. It is the basis for the nation’s constitutional biculturalism and establishes principles for Māori/non-Māori relationship and conduct.
