# Supplementary material for: Student outcomes in a student-led clinic: beyond clinical learning
Source: Front Health Serv. 2026 Oct 9;6:1884776. doi: 10.3389/frhs.2026.1884776 (PMC13690954; doi:10.3389/frhs.2026.1884776)
Supplement: Supplementary file 1 [file Table1.docx]

**Supplementary Figure 1.** Te Kotahi Oranga Student-assisted clinic student survey

**Your consent**

Before we start, we need to confirm that you agree to participate in this survey. Please read the following and click ‘Yes’ if you are happy to continue with the survey.

I understand and agree that:

- I have read the above information about the survey
- I am 16 years or older and can provide consent to participate
- The survey is entirely voluntary and I may choose to stop at any time
- My deidentified survey response will be collected and securely lodged with the Huber Social database My responses are confidential and individuals will never be identified in any reporting; however, group results may be made public.

Please click ‘YES’ to indicate your consent and start the survey. *****

**A little about you**

2. Right now I am feeling...

*(Select only ONE the image you feel closest to)*

3. Please enter your student ID
4. When are you completing this survey? *****
5. Which centre are you enrolled in?
6. What is your programme of study?
7. What is your year of study in your current programme?
8. How do you describe your gender? Please select all that apply:
9. What is your age?

**Overall wellbeing**

The following section asks about your **experiences in life and your overall wellbeing.** There is no wrong answer, just your own observations on how life is for you at this moment.

You will be asked to respond to a number of statements on a 7-point scale. **Please select the position on the scale that best reflects how you feel about that statement.** You can either click and drag the circle on the left-hand side of the scale or click a point directly on the scale line.

Please respond based on how you have been feeling lately, rather than how you have been in the past or how you might like to feel in the future. **Note that the scale may change depending on the statement**, so it is important to read the entire statement and the scale labels.

If any of these questions cause you distress or raise thoughts that you would like to discuss with a professional, either speak with your medical provider or you can click here now for information on free mental health helplines in Aotearoa New Zealand.

You can also **call or text [ ] for free** any time, 24 hours a day to speak with a trained counsellor or contact Lifeline at [ ] or text HELP [ ] for free, 24/7, confidential support - 24 hours a day, 7 days a week.

Health Services:

To make an appointment to see a Health Services nurse, doctor, or counsellor, please phone [ ].

For medical queries or advice, you can email [ ] Please do not use the Health Services email address to make an appointment.

15. In most ways my life is close to my ideal (1. Strongly disagree - 7. Strongly agree)
16. The conditions of my life are excellent
17. I am satisfied with my life
18. So far, I have gotten the important things I want in life
19. If I could live my life over, I would change almost nothing

**Clinical skills**

20. I am confident that I have the knowledge and skills necessary for providing quality clinical care
21. I have had access to learning opportunities which allowed me to develop my clinical knowledge in community care
22. I know what steps I should take to build good relationships with my clients
23. I am confident in my ability to communicate effectively with clients and their whānau
24. I feel that I can handle many things at a time, such as coursework, clinical duties, home care responsibilities, etc.
25. When confronted with a problem, I can find a solution that works
26. I can adapt when unexpected events happen

**Interprofessional teamwork skills and cultural understanding**

15. I believe that working with an interdisciplinary clinical team leads to better outcomes for clients
16. I am able to build and maintain positive relationships with a clinical team
17. I can confidently and clearly express myself to my clinical team
18. I can help two colleagues on my clinical team who are in in conflict reach a peaceful solution
19. My opinions are heard and respected by my clinical team peers
20. I consider the experiences and perspectives of my clients when interacting with them (i.e., sharing a diagnosis, explaining treatment options, etc.)
21. I feel valued and appreciated by my clinical team peers

**Ngā manukura (leadership)**

22. I am able to make decisions about client care confidently and effectively
23. When appropriate, I am comfortable leading a team of my peers in client care management
24. I have had the opportunity to lead client care
25. I am aware of my personal strengths and skills which I would like to continue improving
26. Within my clinical team, I am able to advocate for myself and my client
27. My belief in myself can help me get through difficult times

**Sense of connection and purpose**

28. I feel a sense of belonging with my clinical placement cohort
29. I can see myself as a professional in the health and wellbeing sector
30. I know a healthcare professional I feel comfortable turning to for guidance as I navigate my career path
31. Through my clinical work, I feel a sense of connection with the community (i.e., the community that uses the clinic)
32. My clinical work gives me a sense of purpose
33. I have the opportunity to give back to my community
34. I feel motivated to pursue a career in my healthcare field
35. I feel motivated to pursue a career with underserved communities
36. I am able to set and achieve my own career goals

**My holistic health**

37. I like who I am
38. On most days I get enough sleep and feel well rested
39. In general, my physical health is good
40. I understand how my lifestyle choices, including sleep, hygiene and exercise, affect my health
41. I enjoy life and have fun
42. In the past month, how often have you felt stressed or overwhelmed? (1. A few times a month - 7. Daily or almost daily)
43. In the past month, how often have you felt happy? (1. A few times a month - 7. Daily or almost daily)
44. I feel connected to my cultural identity (1. Strongly disagree - 7. Strongly agree)
45. A te ao Māori perspective is well-integrated into my discipline’s curriculum
46. I feel a connection to our whenua (land)
47. I have a strong support network of family, whānau and/or friends

**Access to resources, services and self-development**

48. I am determined to achieve my goals
49. I have enough income to cover my everyday needs
50. I have access to safe and stable housing
51. My home satisfies my personal needs (i.e., is comfortable, clean, warm, private, etc.)
52. I have access to transport that gets me where I need to go
53. I have access to affordable childcare
54. The career development opportunities I have access to are appropriate for my career goals and interests
55. If needed, I know where I can go for guidance and support to help me navigate my academic journey
56. I have opportunities to learn and participate in cultural activities that are important to me
57. I feel free to be myself around others

**Additional demographic details**

In this last section of the survey, we will be asking you for some additional information about who you are. We collect this type of data so that it can be used to inform our overall results, and so that we can better understand the unique relationships between Te Kotahi Oranga and student practitioners.

**Your survey results are anonymous and individual responses will never be shared; you may skip any question you do not feel comfortable answering.**

67. What is your ethnicity? Please provide up to two ethnicities only:
68. On average, how long is your commute to Te Kotahi Oranga clinic?
69. How many financial dependents do you have?
70. Do you identify as someone currently living with a disability?

*We are asking this question so that we can better support the wellbeing needs of all health ākonga practicing with Te Kotahi Oranga and ensure an equitable experience for all. If you would like more information about accessibility support at Wintec, please visit Student Accessibility Services.*

71. What aspect(s) of your experience in your placement did you find most helpful or beneficial to you and your professional development?
72. What aspect(s) did you find most difficult about your placement experience?
73. How has your placement had an impact on your future practice or career aspirations?
74. Do you have any additional feedback for staff?

**Thank You!**

**Your time, effort and honesty are appreciated.**

If you would like more information on other Wintec/Te Pūkenga services, please see Support Services.

If you would like more information about this survey and how the information will be used, please contact us at: info@hubersocial.com.au

Copyright Huber Social ©
